# Pharmacokinetic modeling of hepatospecific MRI contrast agent flux in large animal models by dynamic contrast-enhanced MRI

**DOI:** 10.21203/rs.3.rs-8176661/v1

**Published:** 2026-01-14

**Authors:** Matthew T. Latourette, Jeremy M.-L. Hix, Jie Huang, Christiane L. Mallett, Kirk A. Muñoz, Erik M. Shapiro

**Affiliations:** 1 Department of Radiology, Michigan State University, East Lansing, MI, USA; 2 Department of Physiology, Michigan State University, East Lansing, MI, USA; 3 Department of Chemical Engineering and Material Science, Michigan State University, East Lansing, MI, USA; 4 Department of Biomedical Engineering, Michigan State University, East Lansing, MI, USA; 5 Department of Small Animal Clinical Sciences, Michigan State University, East Lansing, MI, USA; 6 Institute for Quantitative Health Science and Engineering, Michigan State University, East Lansing, MI, USA^6^

**Keywords:** DCE-MRI, pharmacokinetics, translational imaging, large animal models, hepatic function

## Abstract

**Purpose:**

Evaluate the feasibility of using dogs and a pig as translational models for assessing hepatic flux of hepatospecific gadolinium-based contrast agents using dynamic contrast-enhanced MRI (DCE-MRI).

**Procedures:**

DCE-MRI was performed with hepatospecific agents, gadoxetate disodium (Gd-EOB-DTPA) and gadobenate dimeglumine (Gd-BOPTA), and non-hepatospecific agents. Two validated pharmacokinetic models were applied: a single-input reference region model and a dual-input model using arterial and venous blood signals.

**Results:**

In dogs, hepatic enhancement rose rapidly and plateaued after Gd-EOB-DTPA administration, with minimal decline over an hour. In the pig, both hepatospecific agents showed a rapid rise, blunt peak, and gradual decline. The single-input model revealed significantly higher uptake rates for hepatospecific agents, confirming its sensitivity to hepatocyte uptake. The dual-input model effectively distinguished contrast agent dynamics in dogs and showed promise in the pig.

**Conclusions:**

In this feasibility study, canine Gd-EOB-DTPA pharmacokinetic values closely mirrored those observed in humans. These results establish baseline model parameters in a large-animal setting and support further, more comprehensive investigations into their translational potential for assessing hepatic function using DCE-MRI.

## Introduction

Chronic liver diseases are responsible for substantial mortality and morbidity throughout the world, annually causing approximately two million deaths globally and, in the U.S. alone, $32.5 billion in associated healthcare costs.^1^ Biopsy, the gold standard clinical test, is not suitable for screening or for longitudinal surveillance of liver disease because it is invasive and poses significant risks to the patient. Other clinical tests typically lack specificity and are insensitive to early disease stages, when interventions would benefit patients the most.^2–4^

These limitations have stimulated interest in the potential to noninvasively quantify liver function using dynamic contrast-enhanced MRI (DCE-MRI) with hepatospecific gadolinium-based contrast agents (GBCAs) such as gadoxetate disodium (Gd-EOB-DTPA, trade names Eovist^®^ and Primovist^®^)^4–9^ and gadobenate dimeglumine (Gd-BOPTA, trade name Multihance^®^).^7,8,10,11^ These GBCAs are substrates of the hepatic organic anion transporter polypeptide (OATP) 1B family (OATP1B2 in rodents; OATP1B1 and OATP1B3 in humans) and multidrug resistance-associated proteins (MRPs) expressed by hepatocytes responsible for hepatocyte uptake and clearance, respectively, of substances in the blood.^3,6,12^ Thus, their tissue concentration and functionality are directly coupled with hepatocyte number and physiological function. Gd-EOB-DTPA has received greater research attention because it is more dose-efficient than Gd-BOPTA for probing liver function in humans. Gd-EOB-DTPA is eliminated 50% via the biliary route and 50% renally.^5,7,13^ In contrast, only 2–5% of Gd-BOPTA is eliminated via the biliary route, with the remainder eliminated renally.^7,13^ Additionally, the human hepatocyte uptake rate has been reported to be an order of magnitude greater for Gd-EOB-DTPA than for Gd-BOPTA.^7^

Many studies performed with patients have employed relative liver enhancement^14–16^ and imaged with low temporal resolution scans, interleaving the high spatial resolution scans needed to support clinical care with the late phase DCE-MRI acquisitions.^17^ Increasingly, preclinical studies have employed high temporal resolution DCE-MRI scans, used pharmacokinetic models to quantify the hepatocyte uptake and biliary efflux rates, or both.^4,6,18,19^

Translating methods and results from preclinical rodent experiments to humans is challenging for several reasons. Firstly, preclinical imaging systems lack the panoply of rapid, motion-compensated, 3D imaging sequences of contemporary clinical MRI systems, so experiments cannot be run identically. Secondly, rodent hepatic OATPs and MRPs have different GBCA transport and metabolic rates, making the dynamics very different from humans.^20^ A confounding trait in rodents is that their hepatocytes express OATP1A1, which additionally influxes GBCAs and has no human ortholog.^21,22^ While genetically engineered mice that express human OATP1B1/1B3 on the sinusoidal membrane more closely approximate human liver enhancement for these hepatospecific GBCAs^11^, these OATPs are expressed off of non-native promoters. Thus, diseases that modulate OATP expression through interaction with the promoter cannot be expected to translate. The challenge of simultaneously achieving sufficient temporal resolution to support pharmacokinetic modeling and adequate spatial resolution to measure the dynamic blood signal within small vessels while avoiding partial volume effects also limits rodent models.

Large animal models of disease may provide an important bridge for clinical translation of experimental DCE-MRI.^23,24^ Compared with rodents, the larger vessels in these animals improve the feasibility of individually measuring the vascular inputs to the liver rather than relying upon population-averages or requiring the use of a reference region such as the spleen. However, due to genetic differences between the human OATPs and MRPs and their canine and porcine analogues^25^, it is necessary to first investigate to what extent their hepatospecific GBCA enhancement patterns mimic human liver enhancement before relying on these models for clinical translation. Groups have imaged canine livers with Gd-EOB-DTPA but without estimating pharmacokinetic parameters.^26–30^ Borusewicz *et al* reported that, in dogs, 70% of the administered dose of Gd-EOB-DTPA is eliminated via the bile and the remaining 30% is excreted renally.^26^

To evaluate whether dogs and/or pigs are appropriate models for translational imaging studies, we performed DCE-MRI with Gd-EOB-DTPA and with the non-hepatospecific GBCA gadobutrol (Gd-BT-DO3A, trade name Gadavist^®^) on dogs that were genetic carriers of Glycogen Storage Disease Type IIIa (GSD III) and born with cleft palate, which was surgically corrected at 6 weeks of age, but otherwise phenotypically normal. We also imaged a normal pig multiple times with both Gd-EOB-DTPA and Gd-BOPTA and once with the non-hepatospecific GBCA gadopentetate dimeglumine (Gd-DTPA, trade name Magnevist^®^). We fit two different, previously validated, pharmacokinetic models to the imaging data, a reference region model using the spleen as the surrogate for the vascular input functions^4,31^ and a dual-input model using the abdominal aorta and portal vein as its inputs.^6,18^

## Materials and Methods

### Animal Care

Animals were provided with water *ad libitum* and received portioned feed twice daily. Their environment was maintained at 22° C (±2° C) and 30–70% relative humidity with a standard 12 h light/dark cycle.

This study was intentionally designed as a *feasibility investigation* rather than a population-level statistical trial. Repeated imaging sessions within subjects and across a small number of well-characterized large animals provided sufficient independent observations to assess reproducibility and model stability. Each animal served as its own internal control, maximizing data yield per subject and aligning with internationally recognized ethical frameworks—the 3Rs (Replacement, Reduction, and Refinement)—that govern responsible animal use. All animals were physiologically healthy with normal hepatic function, providing valid translational baselines for human comparison.

A single 2.5-month-old, 25 kg Yorkshire/PIC327 Crossbred male pig was purchased from MSU Swine Farm and single-housed. No adverse events, injuries, or infections were encountered during the course of animal housing. Following the final MRI scan, the pig was euthanized while under deep anesthesia (5% sevoflurane vaporized in 1–2 L/min oxygen) via an intravenous (IV) bolus injection of Euthasol^®^ (390 mg/mL pentobarbital sodium and phenytoin sodium solution) at a minimum dose of 0.1 mL/kg BW. Death was confirmed by thoracic auscultation of cardiac arrest.

Three dogs (two females ~4 years old; one male ~6.5 years old) that are genetic carriers of GSD III were socially housed in dog kennel runs twice the standard minimum housing requirements to provide space for socializing and exercise, with toys, manipulanda, regular staff interaction, and outside walking sessions for enrichment. Before recovery from the final MRI scan, three ultrasound-guided Tru-Cut^®^ liver biopsies were obtained. Following anesthesia recovery, the dogs were transferred back to the College of Veterinary Medicine (CVM) until they were each successfully adopted to caring families.

Food was withheld overnight (8–12 h) prior to scheduled imaging sessions. Water was provided until the sedative was administered. Each GBCA used was FDA-approved for human use and administered at the body weight-normalized dosage specified in the manufacturer’s prescribing guidelines (0.025 mmol/kg for Gd-EOB-DTPA, 0.1 mmol/kg for Gd-BOPTA, 0.1 mmol/kg for Gd-BT-DO3A, 0.1 mmol/kg for Gd-DTPA). Experiments were staggered by 1 or 2 weeks to ensure elimination of the agent between sessions and for scheduling considerations.

### Swine Study Anesthesia and MRI Protocol

For the first session, the pig was lightly sedated via intramuscular Telazol (4 mg/kg) in the vivarium and carted to the MRI suite for induction of general anesthesia and intubation. Transportation reduced the level of sedation of the pig, so for subsequent scan sessions the animal was sedated, anesthetized, and intubated in the vivarium, and then transported under inhalant anesthesia to the MRI. Bilateral ear vein catheters were installed in the vivarium and blood was collected pre- and post-MRI for biochemistry and complete blood count (CBC) analyses. Lidocaine, to blunt the gag reflex, (1 mg/kg) and propofol (4 mg/kg titrated to effect) were administered intravenously for induction of general anesthesia and to facilitate intubation of the pig.

During MRI, the pig was positioned in sternal recumbency and was breathing well spontaneously while being maintained under anesthesia (2–3% sevoflurane in 100% oxygen). IV Fluid Therapy (0.9% NaCl for Injection, USP) was given as a continuous drip (5–10 mL/kg/h) during imaging. Vital signs were stable and continuously monitored throughout anesthesia, which included heart rate, blood pressure (BP), pulse-oximetry, core body temperature (CBT), end-tidal CO2, and echocardiogram. Oscillometric BP was measured via external peripheral limb with an inflatable cuff. CBT was measured via rectal thermometer pre- and post-MRI. During the second session, the pig exhibited hyperthermia at the end of the experiment. Consequently, in subsequent sessions, a cooling recirculating water pad was placed ventrally under the animal and use of a plastic cover for the scanner bed, employed for the first and second sessions, was discontinued. These mitigations maintained stable, normothermic CBT subsequently.

### Canine Study MRI Protocol

Each dog was sedated with butorphanol (0.2–0.4 mg/kg IM). Under sedation, a unilateral peripheral IV catheter was placed in the cephalic vein and secured. Artificial Tears were applied to both eyes to prevent corneal drying or abrasions. Anesthesia was induced with propofol (7 mg/kg IV, titrated to effect) before the animal was intubated with an appropriately sized endotracheal tube, and the tube secured in place. Dogs were maintained under general anesthesia with sevoflurane (2–4%) in 100% oxygen (1 L/min). The animal was positioned in sternal recumbency and was breathing well spontaneously while inside the MRI bore. The vital signs monitored were as described in the swine protocol. The animal received supplemental IV fluids (0.9% NaCl for Injection, USP or Lactated Ringers Solution, USP) (5 mL/kg/h) during the procedure. Blood samples were collected pre- and post-MRI for biochemistry and CBC analyses.

### Imaging Swine Study

The pig underwent five separate 60-minute DCE-MRI sessions on a Siemens Esprit 1.5 T MRI over the course of six weeks, each time injected with a different contrast agent, with details recorded in Table 1. Scans were conducted at least one week apart to ensure contrast agent clearance. The FOV was increased for the last scan because of the pig’s growth. MRI acquisition parameters are shown in Table 3.

### Canine Study

Three dogs were injected with a GBCA bolus and imaged with a Siemens Espree 1.5 T MRI multiple times, with details recorded in Table 2. Scans were spaced at least one week apart to ensure clearance of the contrast agent. For each dog, three scans with the hepatospecific GBCA Gd-EOB-DTPA and one scan with the extracellular fluid GBCA Gd-BT-DO3A were included in the study. Dog 1 was imaged once before beginning the study to determine the MRI acquisition parameters needed for the study, however, this scan was excluded from the analysis. One scan from dog 2 was also excluded due to an equipment failure during imaging. A repeat scan was conducted to replace it. The pre-MRI hematocrit measurement was used for pharmacokinetic analyses. MRI acquisition parameters are shown in Table 3.

### Image Registration

AFNI (RRID:SCR_005927) was used to correct for subject motion for all DCE-MRI series.^32,33^ Each 3D volume’s images were aligned to the first volume’s images with the *3dvolreg* routine, and Fourier interpolation was used for the alignments.

### Image Segmentation

3D maximum, mean, and standard deviation intensity projection images were computed by projecting the relevant measure (maximum, mean, or standard deviation) along the temporal dimension of the DCE-MRI series for each spatial location. These images were then segmented with MATLAB’s Medical Image Labeler using automatic boundary tracing, morphological operations, thresholding, and manual refinement of the region of interest (ROI) boundaries.

Large ROIs encompassing liver and spleen parenchyma were drawn on the mean intensity projection images because these projections diminish the appearance of respiratory motion artifacts. Large vessels, edges where partial volume effects are likely, and slices at the ends of the 3D volume were excluded from the organ ROIs. Liver ROIs were cross referenced with the SD intensity projection images to identify and exclude the bile ducts. The spleen was not present in the imaged volume for the last scan of the pig, precluding use of the TRISTAN model for its analysis.^31^

Abdominal aorta and portal vein ROIs were drawn on the maximum intensity projection images because they accentuate the contrast between vasculature and surrounding tissue. Vascular ROIs were further refined by performing morphological dilation with a radius of one pixel, ranking the ROI’s pixels in order by signal intensity, and thresholding to retain the top third of pixels by rank to exclude extraluminal pixels and pixels affected by partial volume effects.

### Noise Filtering

Signals extracted from the ROIs were filtered to attenuate noise. The filtering scheme blended two median filters of differing kernel sizes, applying minimal filtering during the initial concentration peak and greater noise attenuation in the late phase where concentration changes slowly. Computations were performed using MATLAB’s *smoothdata* function with the *movmedian* algorithm.

Filter transition points were approximately five minutes and 30 minutes after the contrast bolus injection. The size of the filter kernel was three from the beginning to the first transition point and seven from the second transition point through the end of the acquisition. Between the transition points, the filtered signal was computed as a weighted sum of the two median filtered signals with weights changing linearly between zero and one, gradually increasing noise attenuation until the second transition point.

### Signal Intensity to Concentration Conversion

MR signal intensity was converted to gadolinium concentration using the following equations:

[1] $$a=\frac{S(t)}{S(0)}\cdot \frac{1-exp\left(-R_{1}(0)\cdot T_{R}\right)}{1-cos\;\theta \cdot exp\left(-R_{1}(0)\cdot T_{R}\right)}$$

[2] $$R_{1}(t)=-\frac{ln\left(\frac{1-a}{1-cos\;\theta \cdot a}\right)}{T_{R}}$$

[3] $$C_{e}(t)=\frac{R_{1}(t)-R_{1}(0)}{r_{1,\mathrm{spleen}}v_{e,\mathrm{spleen}}}$$

[4] $$C_{i}(t)=\frac{C_{t}(t)-v_{e,\mathrm{liver}}\cdot C_{e}(t)}{1-v_{e,\mathrm{liver}}}$$

[5] $$C_{t}(t)=\frac{R_{1}(t)-R_{1}(0)}{r_{1,\mathrm{tissue}}}$$

where $R_{1}(0)$ is the pre-contrast longitudinal relaxation rate for the tissue; $R_{1}(t)$ is is the longitudinal relaxation rate at $t;\;T_{R}$ is the repetition time; θ is the flip angle; S(0) is the mean baseline pre-contrast signal intensity of the ROI; S(t) is the mean signal intensity of the ROI at time $t;\;C_{e}$ is the concentration in the extravascular extracellular space (EES); $C_{t}$ is the liver concentration; $C_{a}$ and $C_{v}$ are the arterial and venous blood concentrations, respectively, computed in the same manner as $C_{t};r_{1,\mathrm{tissue}}$ is the *in situ* relaxivity of the contrast agent; $v_{e,\mathrm{tissue}}$ is the EES volume fraction of the relevant tissue.^5,34,35^ Published rat EES volume fractions of 0.23 and 0.43 were used for the liver and spleen, respectively, assuming that these values do not differ appreciably between mammals.^5^ For non-hepatospecific agents, the intracellular concentration in liver was assumed to be zero ($C_{i}(t)=0$). Constant $T_{1}(0)$ values were used for each tissue for all analyses $\left(R_{1}=1/T_{1}\right)$: 581 ms for liver, 1172 ms for spleen, 1480 ms for blood.^36,37^ Haacke *et al* showed that using fixed values for $R_{1}(0)$ reduces the influence of noise on the concentration time integral.^34^ *In situ* relaxivity $\left(r_{1}\right)$ values used for Gd-EOB-DTPA in the dog study were: 14.6 s^−1^·mM^−1^ in liver^31^ and 7.3 s^−1^·mM^−1^ in blood and spleen.^38^ Values used for Gd-BT-DO3A in the dog study were: 5.2 s^−1^·mM^−1^ in liver and 5.3 s^−1^·mM^−1^ in blood and spleen.^38^ *In situ* relaxivity values used for Gd-EOB-DTPA in the pig study were: 14.6 s^−1^·mM^−1^ in liver^31^ and 6.9 s^−1^·mM^−1^ in blood and spleen.^39^ Values used for Gd-BOPTA in the pig study were: 6.3 s^−1^·mM^−1^ in liver and 6.7 s^−1^·mM^−1^ in blood and spleen.^38^ Values used for Gd-DTPA in the pig study were: 3.4 s^−1^·mM^−1^ in liver^40^ and 4.3 s^−1^·mM^−1^ in blood and spleen.^38^ When canine and/or porcine literature values were unavailable, the most closely related mammal (bovine, human, or rat) value available was used.

### Model Fitting

Pharmacokinetic models were fit using MATLAB’s *MultiStart* global optimizer, which performs repeated fitting using a local optimizer (*fmincon*) with many different starting estimates, returning the best overall fit. Starting estimates were pseudorandom numbers covering the range of expected variation for both normal and abnormal subjects. To ensure reproducibility, the Mersenne Twister pseudorandom number generator’s seed value was reset each time before generating the starting estimates. Two types of starting estimates were generated for each model fit: “narrow range” estimates encompassing the expected values for normal liver and “wide range” estimates spanning several orders of magnitude to account for abnormal uptake, efflux, fibrosis, etc. in diseased liver.

Pharmacokinetic models were fit four times per DCE-MRI scan, using both least squares (LSQ) and least absolute residuals (LAR) fitness criteria, with and without noise filtering. The relevant metrics were:

[6] $$p_{LSQ}=\underset{p}{argmin}\left(\frac{1}{2n}\cdot \|r{\|}_{2}\right)=\underset{p}{argmin}\left(\frac{1}{2n}\cdot \sqrt{r\cdot r^{T}}\right)$$

[7] $$p_{LAR}=\underset{p}{argmin}\left(\frac{1}{2n}\cdot \|r{\|}_{1}\right)=\underset{p}{argmin}\left(\frac{1}{2n}\cdot \sum_{m=1}^{n}\left|r_{m}\right|\right)$$

Where $r=C-\hat{C}$ is the residual vector and $r^{T}$ its transpose; C is the concentration vector of the fitted model; $\hat{C}$ is the concentration vector obtained from the ROI mean signal intensity using the conversion described above; $p_{LSQ}$ and $p_{LAR}$ are the fitted model’s parameter vectors with LSQ and LAR fitness criteria, respectively; p is the model’s parameter vector; and n is the number of DCE image acquisitions.

Computations were performed on a Dell Precision 5570 computer with an Intel Core i7–12700H processor with 14 cores running at 2.3 GHz and 64 GB RAM on Microsoft Windows. Custom MATLAB^®^ software was written for the analysis.^41^ A parallel pool of 14 MATLAB workers was employed. Model fitting was constrained to ≤ 3000 iterations or 20 minutes per run of the local optimizer, but all runs required < 1 minute of computing time in practice, excluding the time to initialize the parallel pool.

### TRISTAN Model

#### Starting Estimates

For each optimization, 20 “narrow range” and 80 “wide range” pseudorandom starting parameter vectors were generated. The “narrow range” starting estimates were uniformly distributed on the interval [0.001, 0.05] s^−1^ for $k_{1}$ and [0.00001, 0.002] s^−1^ for $k_{2}$. The “wide range” starting estimates for $k_{1}$ and $k_{2}$ were logarithmically distributed on the interval [10^−10^, 1] s^−1^.

#### Constraints

Model parameters $k_{1}$ and $k_{2}$ were constrained to the range [eps, 1.0] s^−1^, where $eps=2^{-52}\approx 2.22\times 10^{-16}$.

#### Equations


[8] $$v_{h}\frac{dC_{i}}{dt}=k_{1}C_{e}(t)-k_{2}C_{i}(t)$$

[9] $$C_{i}(t[n])=\left(exp\left(-k_{2}\cdot t[n]/v_{h}\right)\;*\;\frac{k_{1}C_{e}(t[n])}{v_{h}}\right)\cdot \Delta t$$

[10] Δt=t[n+1]-t[n]

where $v_{e}$ is the EES volume fraction, $v_{h}=1-v_{e}$ is the hepatocyte volume fraction, $C_{i}$ is the intracellular concentration, t[n]∈R is the time relative to the bolus injection for sample n∈N of the DCE series (t=0 at the time of bolus injection), $k_{1}$ is the influx rate, $k_{2}$ is the efflux rate, $C_{e}$ is the EES concentration, Δt is the temporal resolution, and ∗ is the discrete convolution operator.^31^ The scaling term Δt was appended to ensure the discrete-time results computed via Eq. 9, which is a closed-form solution to Eq. 8, would agree with results obtained using continuous-time methods to solve the differential equation.

#### Berks (Biexponential) Model Starting Estimates

500 “narrow range” and 500 “wide range” pseudorandom starting parameter vectors were generated per optimization. The “narrow range” starting estimates were uniformly distributed on the interval [0.0001, 1.0] s^−1^ for $\alpha_{+},\;\alpha_{-},\;\beta_{+}$, and $\beta_{-}$ and [0.05, 0.3] for $f_{a}$. The “wide range” starting estimates for $\alpha_{+},\;\alpha_{-},\;\beta_{+}$, and $\beta_{-}$ were logarithmically distributed on the interval [10^−10^, 1] s^−1^. The “wide range” starting estimates for $f_{a}$ were uniformly distributed on the interval [0.0, 0.6].

#### Constraints

Model parameters $\alpha_{+},\;\alpha_{-},\;\beta_{+},\;\beta_{-}$, and $f_{a}$ were each constrained to the range [eps, 1.0] s^−1^, where $eps=2^{-52}\approx 2.22\times 10^{-16}$.

#### Equations


[11] $$C_{p}(t)=\frac{f_{a}\cdot C_{a}(t)+\left(1-f_{a}\right)\cdot C_{v}(t)}{1-Hct}$$

**Figure 1.**
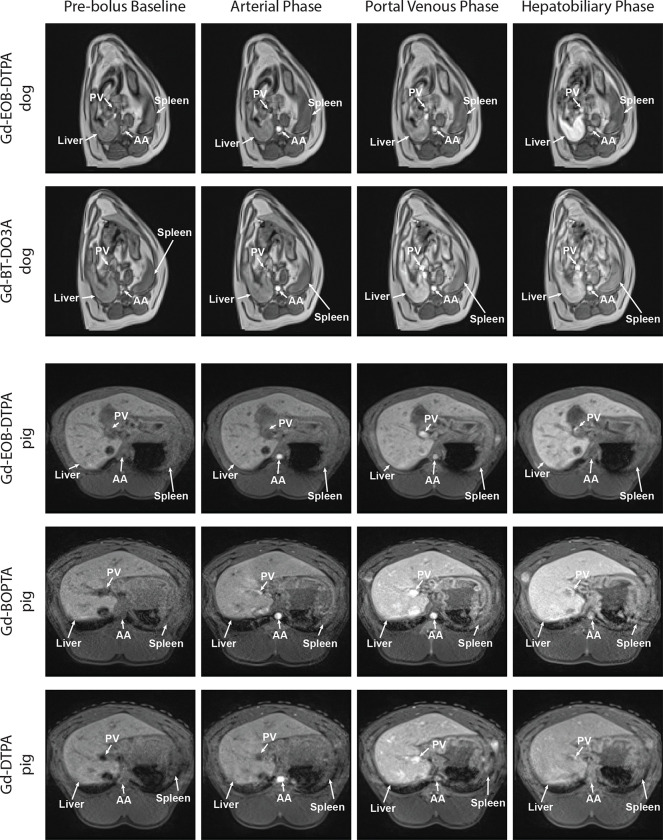
Pharmacokinetic Model Diagrams Diagrams of the mass transport flows and compartments for the single input two-compartment reference region TRISTAN model and the dual-input two-compartment biexponential Berks/Georgiou model employed in this study are shown. $F_{p}$ is the total blood flow, $v_{e}$ is the volume fraction of the EES compartment, $v_{i}$ is the volume fraction of the intracellular space (hepatocyte) compartment, $k_{1}$ and $k_{i}$ are the influx rate constants, $k_{2}$ and $k_{\mathrm{ef}}$ are the efflux rate constants, $f_{a}$ is the arterial flow fraction, and $f_{v}=1-f_{a}$ is the venous flow fraction.

[12] $$C_{t}(t[n])=\left(\alpha_{+}\cdot exp\left(-\beta_{+}\cdot t[n]\right)+\alpha_{-}\cdot exp\left(-\beta_{-}\cdot t[n]\right)\right)\;*\;C_{p}(t[n])\cdot \Delta t$$

where $C_{p}$ is the plasma concentration, $f_{a}$ is the arterial flow fraction, $C_{a}$ is the arterial blood concentration, $C_{v}$ is the venous blood concentration, Hct is the hematocrit, $C_{t}$ is the liver tissue concentration, t[n]∈R is the time relative to the bolus injection for sample n∈N of the DCE series (t=0 at the time of bolus injection), and Δt is the temporal resolution. The α and β values do not have a direct physiological interpretation but may be used to compute physiologic parameters for either the active uptake or passive diffusion regime, as described in Berks *et al*.^18^ Unlike Berks *et al*, our analysis was ROI-based. We did not compute parametric maps. The TRISTAN and Berks pharmacokinetic models’ compartments and flows are shown in Figure 1.

Statistical analysis was done in GraphPad 8.2.0. To compare the model fitting techniques (LAR vs. LSQ and noise filtering vs. no noise filtering), the dog Gd-EOB-DTPA dataset was analyzed using a three-way analysis of variance with matching by factors. Each kinetic parameter was analyzed separately. An unpaired two-tailed t-test with Welch’s correction for unequal variances was used to compare Gd-EOB-DTPA with Gd-BT-DO3A in dogs with matching model fitting techniques. Comparisons of the dogs with the pig were performed on the kinetic parameters fitted using LAR with noise filtering. Each of the Berks model’s kinetic parameters was compared between animals using an unpaired two-tailed t-test, with equal population variance assumed. The threshold for significance was set to *p*<0.05.

## Results

### Imaging

DCE-MRI images of acceptable quality were obtained from three dogs, each scanned three times with Gd-EOB-DTPA and once with Gd-BT-DO3A. Liver, spleen, abdominal aorta, and portal vein were imaged. Five DCE-MRI scans of a pig were performed (two with Gd-EOB-DTPA, two with Gd-BOPTA, and one with Gd-DTPA) with the liver, abdominal aorta, and portal vein present within the FOV. Spleen was absent within the FOV for the second Gd-EOB-DTPA scan, precluding use of the TRISTAN model for its analysis because the model requires the spleen signal as a surrogate for the liver’s EES concentration. The first Gd-BOPTA scan performed on the pig was excluded from the study because the animal became hyperthermic during imaging (core body temperature was 40.1° C immediately before and 41.8° C after MRI) and exhibited rapid efflux not observed in any other scan. Enhancement of the vessels, liver, and spleen was confirmed visually for all scans.

**Figure 2.**
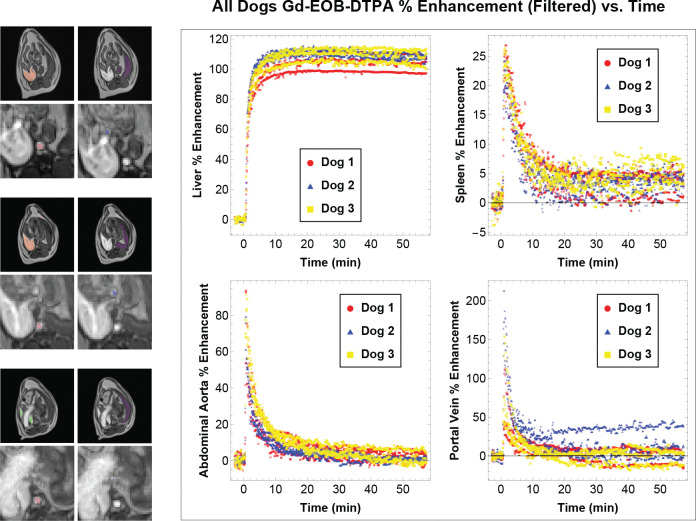

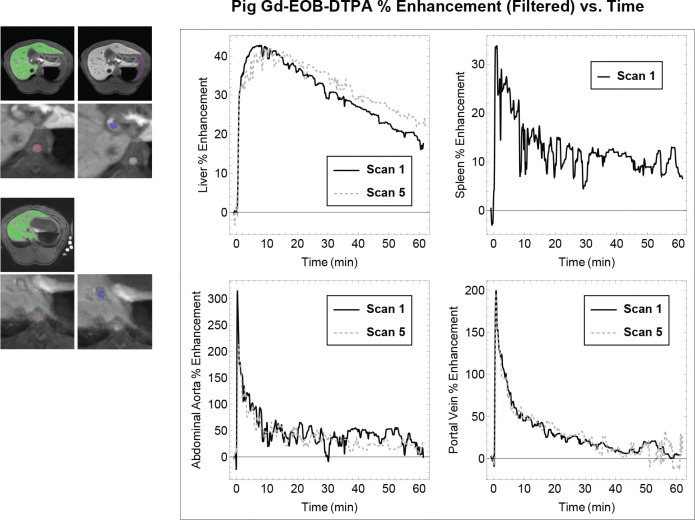

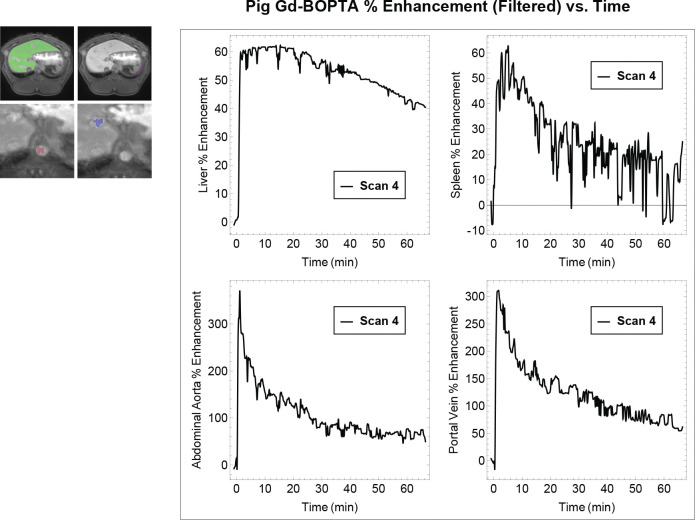

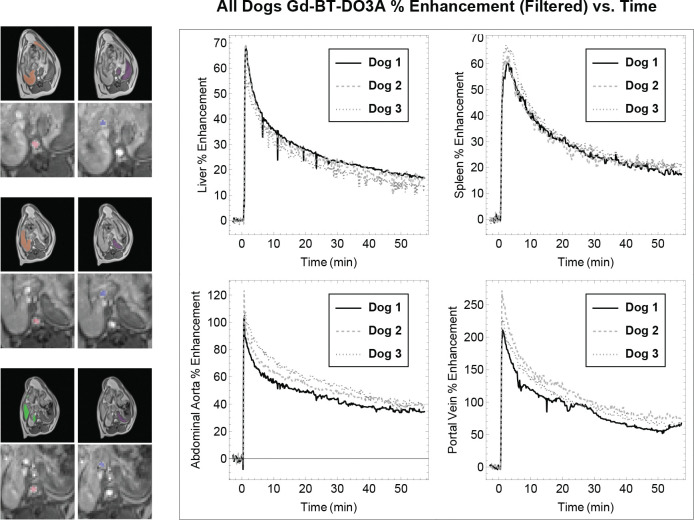

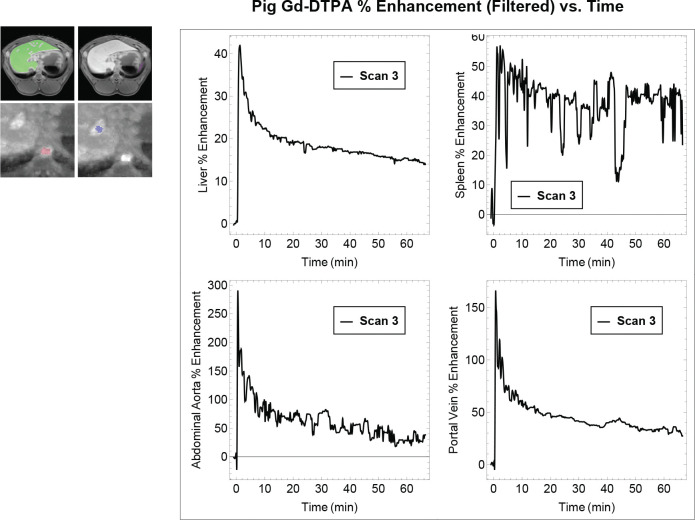
Images In Different Contrast Phases From left to right, the columns portray examples of the pre-contrast baseline, arterial, portal venous, and hepatobiliary phases of the DCE-MRI acquisitions. Each combination of GBCA and species is depicted in a separate row, with dog images presented in the top two rows and pig images presented in the bottom three rows.

Examples of the pre-contrast, arterial, portal venous, and hepatobiliary phases of the DCE-MRI acquisitions are shown for both dog and pig in Figure 2. Although image registration was largely successful in correcting bulk subject motion in the dog and pig scans, some respiratory motion artifacts remained, contributing noise to ROI-derived signals.

### Pharmacokinetics

**Figure 3.**
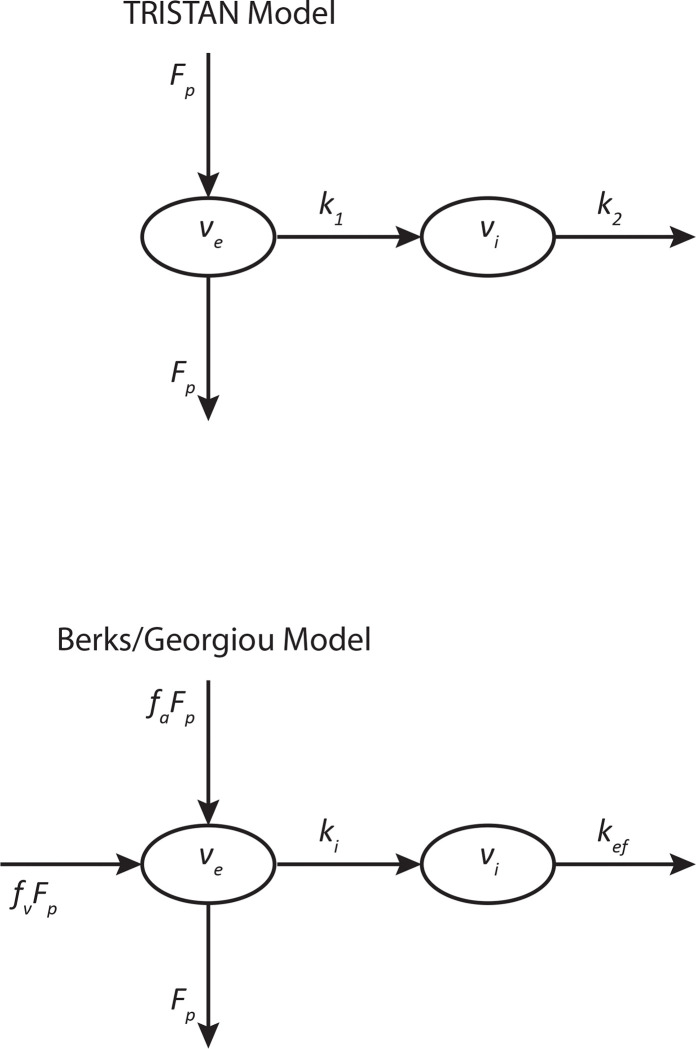
Percent Enhancement — Filtered Data Single slice examples of the ROIs as well as plots of the percent enhancement are shown in five panels. All data in this figure had a noise filter applied. **(a,b)** Display plots for Gd-EOB-DTPA and Gd-BT-DO3A, respectively, in dogs. **(c,d,e)** Display plots for Gd-EOB-DTPA, Gd-BOPTA, and Gd-DTPA, respectively, in the pig. Notably, panel **c** reveals the enhancement pattern remained consistent between the first and the last scan despite the pig doubling in body weight as it grew over the course of the study.

**Figure 4.**
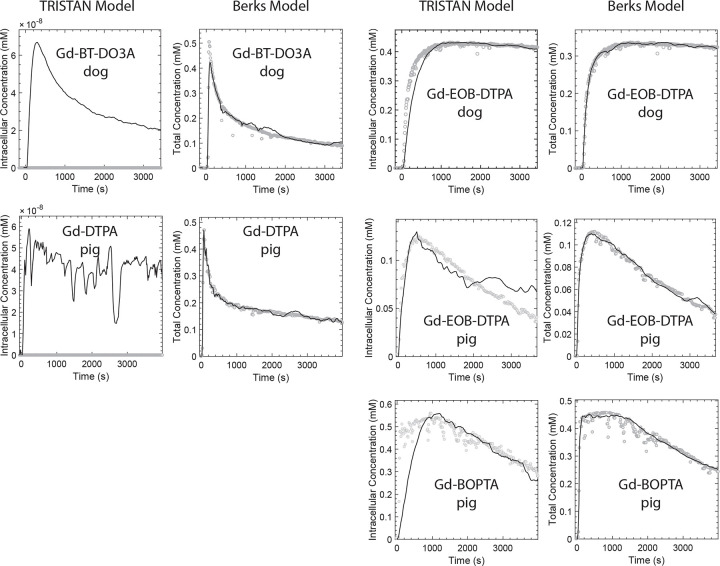
Model Fit Plots Plots depict typical examples of image-derived liver Gd concentration data (orange dots) and corresponding model fits (black line). The two leftmost columns show examples of fitting the TRISTAN (first column) and Berks (second column) models to data obtained with non-hepatospecific GBCAs. The two rightmost columns show examples of fitting pharmacokinetic models to data for hepatospecific GBCAs. The first row shows examples in dogs. The remaining rows show examples in the pig.

In dog liver, Gd-EOB-DTPA’s concentration rose rapidly to a plateau after bolus injection with minimal wash-out during the 1-hour experimental timeframe. In pig liver, the concentration of both Gd-EOB-DTPA and Gd-BOPTA rose rapidly after bolus injection, reached a blunt peak, and decreased steadily thereafter. For the non-hepatospecific agents, Gd-BT-DO3A in the dogs and Gd-DTPA in the pig, the liver tissue concentration rose rapidly from baseline to a sharp peak and then decayed exponentially afterward. Examples depicting these enhancement patterns for the various contrast agents with filtering applied are shown together with single slices of the 3D ROIs in Figure 3. Typical model fits are shown in Figure 4. The complete numerical details for the swine and canine studies are provided in Tables 4 and 5, respectively.

We compared LAR vs. LSQ with and without noise filtering for Gd-EOB-DTPA in dogs and there was no significant difference between parameters for any fitting or filtering method (*p*>0.05). When Gd-EOB-DTPA and Gd-BT-DO3A kinetics were compared in dogs, the difference in $k_{1}$ between contrast agents was significant for all model fitting methods. The difference in $k_{2}$ was statistically significant when using LSQ as the fitness criterion for model fitting. However, the $k_{2}$ result may be spurious, due to a numerically degenerate condition that occurred when fitting the TRISTAN model using LSQ, which resulted in the Gd-BT-DO3A $k_{1}$ and $k_{2}$ values lacking any variance whatsoever. This numerical degeneracy did not occur when the LAR fitness criterion was used to fit the model. The difference in $k_{2}$ between contrast agents did not reach the significance threshold when using LAR. The Berks model’s $\alpha_{+}$ was significantly different between Gd-EOB-DTPA and Gd-BT-DO3A for all model fitting methods; $\beta_{-}$ was significantly different for the LSQ criterion, with or without noise filtering, but not for the LAR criterion. For the Berks model’s $\alpha_{-}$ and $\beta_{+}$, the agents were not significantly different. The difference in $f_{a}$ between contrast agents was significant for all model fitting methods. When the pig and dogs were compared, $\alpha_{+}$ was significantly greater in dogs. $\alpha_{-},\;\beta_{+},\;\beta_{-}$, and $f_{a}$ did not differ significantly between species.

For the TRISTAN model in the dogs and the pig, the initial rise of the modeled Gd-EOB-DTPA or Gd-BOPTA concentration lagged the image-derived concentration in all cases. Nonetheless, the model produced stable estimates of the uptake and efflux rates. The TRISTAN $k_{1}$ uptake rate for Gd-EOB-DTPA in dogs was higher than in the pig while the $k_{2}$ efflux rate was lower. The pig’s TRISTAN $k_{1}$ uptake rates for Gd-EOB-DTPA and Gd-BOPTA were of comparable magnitude, but the $k_{2}$ efflux rate was higher for Gd-EOB-DTPA than for Gd-BOPTA. In both dogs and the pig, the TRISTAN k1 values differed by several orders of magnitude between hepatospecific agents (Gd-EOB-DTPA and Gd-BOPTA) and non-hepatospecific agents (Gd-BT-DO3A and Gd-DTPA). TRISTAN $k_{2}$ values also differed by at least an order of magnitude between hepatospecific agents and non-hepatospecific agents, although the degree of difference varied by fitness criterion.

The mathematical form of the TRISTAN model does not permit representing a straight line with positive, nonzero model parameters. Thus, the TRISTAN model minimized the intracellular concentration to the extent possible within our constraints but did not exhibit a linear shape, as seen in the leftmost panels of the first and second rows of Figure 4.

The choice of LAR vs. LSQ fitness criterion was largely inconsequential to the resulting pharmacokinetic model fits. Likewise, fitting models to unfiltered vs. filtered data rarely had an appreciable effect on the results. The choice of LAR vs. LSQ affected some model parameters when fitting data from scans performed using non-hepatospecific agents Gd-BT-DO3A (dog) and Gd-DTPA (pig), particularly the TRISTAN $k_{2}$ value. The TRISTAN $k_{1}$ and $k_{2}$ values obtained using LSQ for Gd-BT-DO3A in dogs and Gd-DTPA in the pig were identical to at least four decimal places for both filtered and unfiltered data across all animal subjects, a result likely originating from a numerically degenerate case arising during minimization of the objective function. TRISTAN $k_{1}$ and $k_{2}$ values obtained using LAR were unaffected by this issue. Across repeated imaging sessions, the pharmacokinetic parameters in dogs remained stable within each subject and consistent across animals. The reproducibility of these values across independent acquisitions supports the reliability of the model fits despite the limited cohort size and demonstrates that the key physiological trends can be confidently discerned from the data.

**Figure 5.**
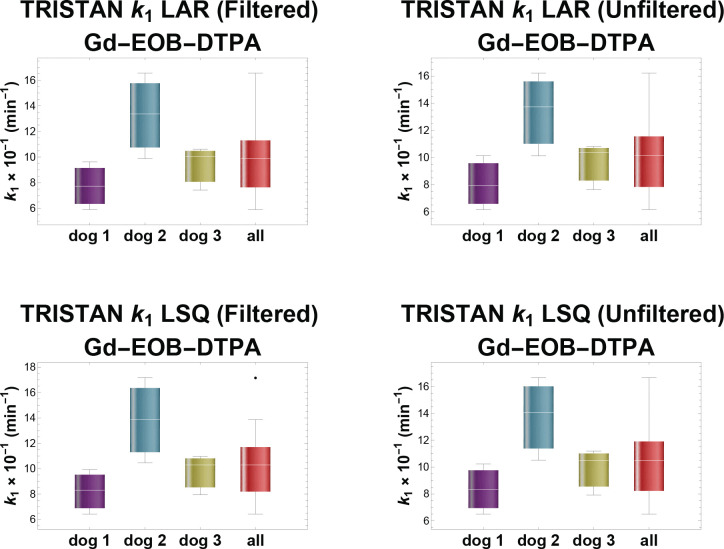

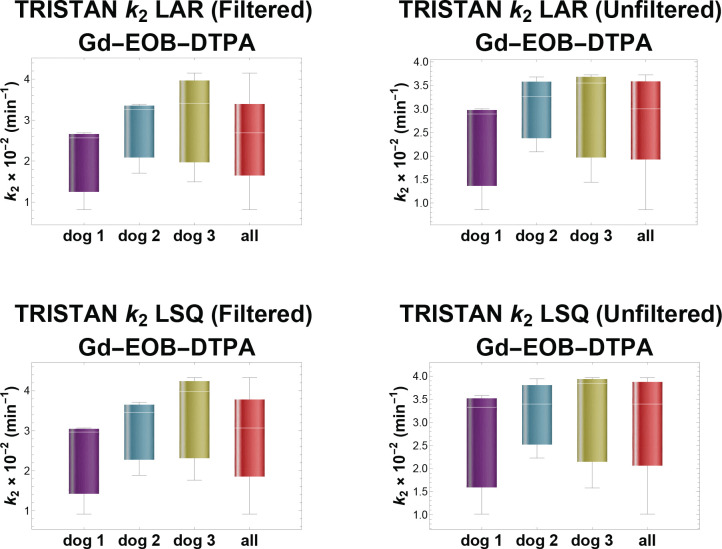

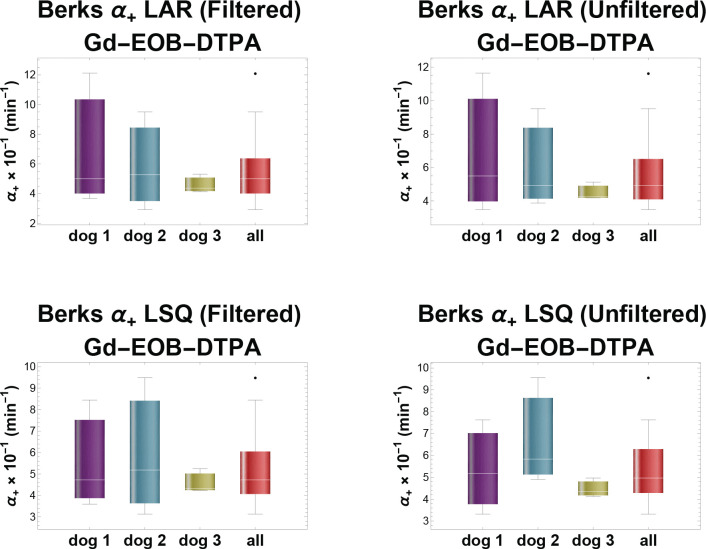

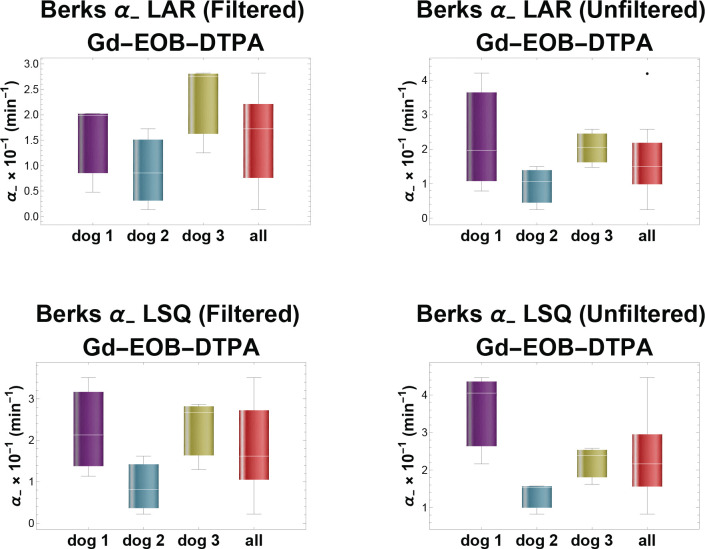

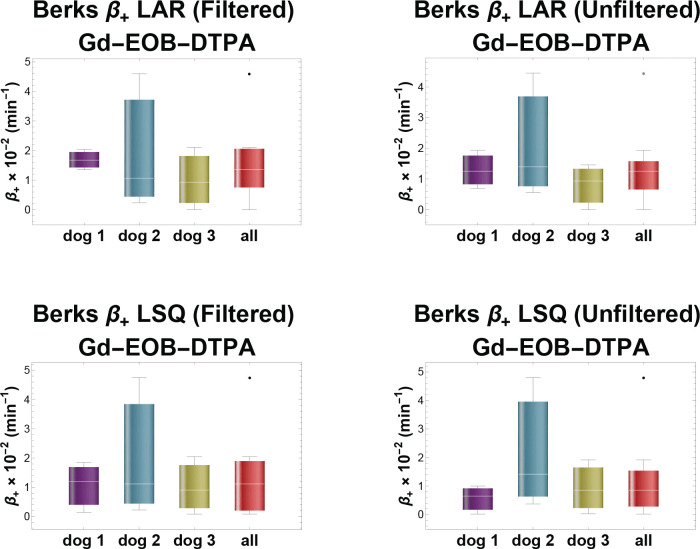

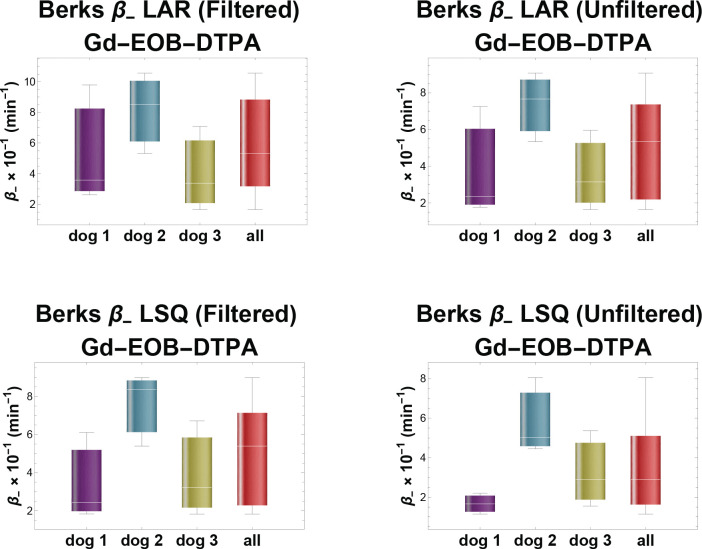

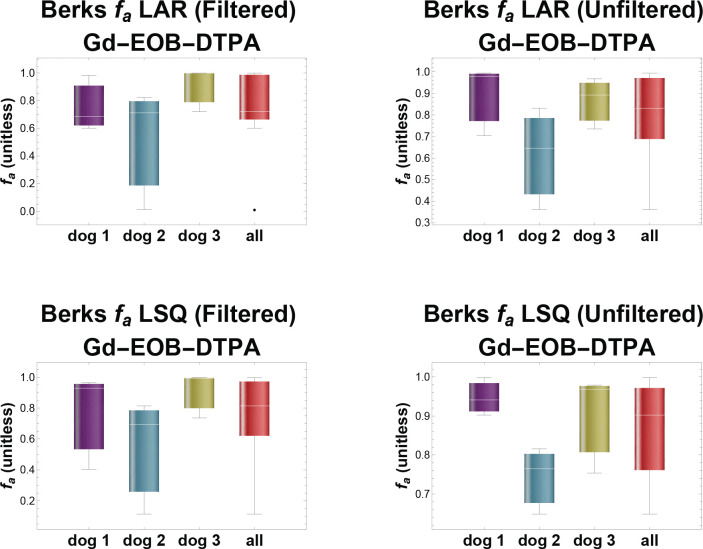

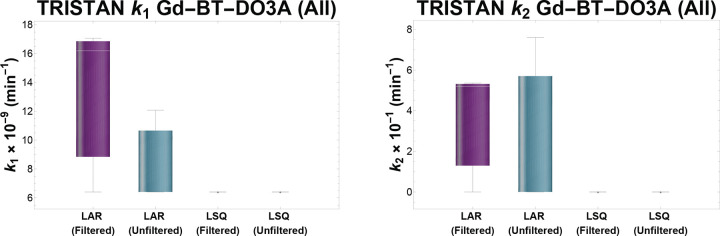

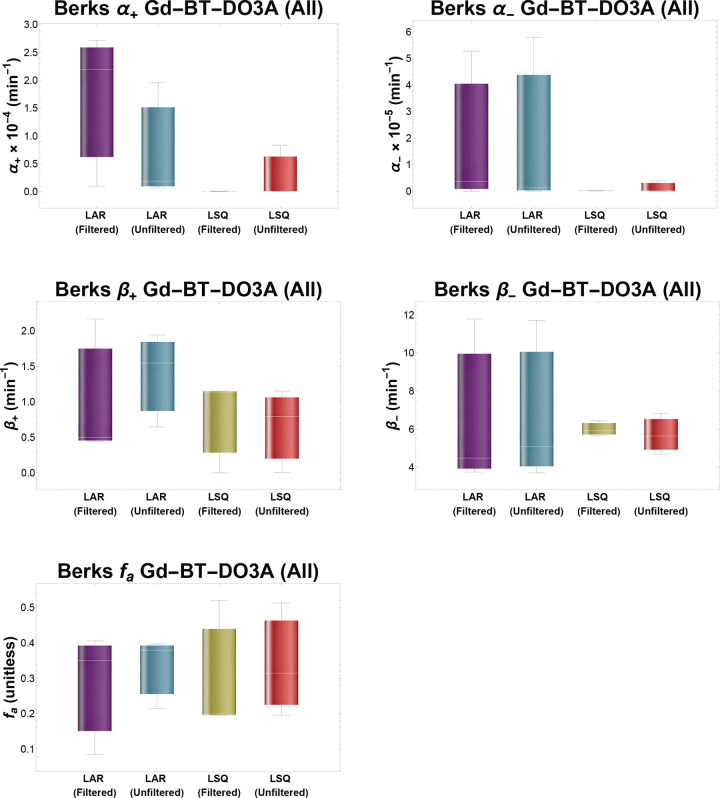
Estimated Model Parameters Box and whisker plots depict the dispersion of the parameter estimates for both pharmacokinetic models, with each GBCA (Gd-EOB-DTPA and Gd-BT-DO3A), and each combination of fitness criterion (LSQ or LAR) and noise filtering (unfiltered or filtered). For Gd-EOB-DTPA, the plots show the data for individuals as well as the dogs in aggregate. The upper and lower bounds of the box represent the third and first quartiles, respectively. The central line inside the box represents the mean. The upper and lower fence markers represent the highest and lowest observations in the data that are within the confidence interval, defined as the mean ± 1.5 times the interquartile range. Dots represent observations outside the confidence interval (outliers). **(a,b)** TRISTAN model with hepatospecific agent, Gd-EOB-DTPA. **(c,d,e,f,g)** Berks model with hepatobiliary agent, Gd-EOB-DTPA. **(h)** TRISTAN model with non-hepatospecific agent, Gd-BT-DO3A. **(i)** Berks model with non-hepatospecific agent, Gd-BT-DO3A.

Individual differences between dogs were observed in the TRISTAN $k_{1}$ and $k_{2}$ values for the Gd-EOB-DTPA scans, with dog 2’s $k_{1}$ elevated relative to the others. Differences between dogs were also observed in the Berks model’s $\beta_{-}$, and $f_{a}$ values. Dog 2’s $\beta_{+}$ range overlapped the others, but its dispersion was greater. Dog 2’s $\beta_{-}$ was the largest. Dog 2’s $f_{a}$ was lower in comparison to the others, particularly when the model was fit to unfiltered data. Most of the $f_{a}$ values overestimated the arterial flow fraction and were outside the physiologic range (0.17 ± 0.12).^6^ Box and whisker plots of the fitted model parameters are shown in Figure 5.

## Discussion

Hepatic enhancement differed substantially between the dogs and the pig. Both species exhibited rapid enhancement, but in dogs it remained constant throughout the entire ~50 minutes post-peak while for the pig, it steadily declined to half the peak value at 60 minutes. In this feasibility study, we found the enhancement in dogs to be a closer match to that of humans than in the pig.^6,42^ Still, determining conclusively which animal species is most analogous to humans is not possible without a paired human study. Nonetheless, it is clear that both dogs and pigs are better suited than rodents, which exhibit much faster efflux of hepatospecific MRI contrast agents than either of these two species.

Although the number of animals in this feasibility study was necessarily limited, validity arises from the number of independent observations and the magnitude of the observed effects rather than from subject count alone. Repeated sessions in individual dogs produced consistent uptake and clearance parameters, and the separation between hepatospecific and non-hepatospecific kinetics was large relative to both intra- and inter-animal variability. In such contexts, reproducibility and effect size drive confidence more than cohort size. These findings establish that even small, rigorously acquired datasets can yield robust pharmacokinetic parameters and confirm the feasibility of large-animal DCE-MRI for translational studies.

As expected, both models had parameters that distinguished between hepatospecific and non-hepatospecific contrast agents. This study established baseline values for these model parameters in phenotypically normal dogs that are genetic carriers of GSD III and a normal pig, laying a foundation for future experiments using these methods to measure liver function. Distinguishing between the uptake of hepatospecific and non-hepatospecific GBCAs is essential for a pharmacokinetic model to be an imaging biomarker of liver function, as the changes in hepatospecific GBCA transport due to liver function will necessarily be smaller than the difference between the uptake of a hepatospecific GBCA and a non-hepatospecific GBCA.

Uptake and efflux rates for Gd-EOB-DTPA obtained via the TRISTAN model were comparable to literature values reported for healthy human subjects. However, there is considerable variation between studies in the reported values for these rates and achieving consistency between sites with different MRI scanners and scan protocols remains an area of active research. Gd-EOB-DTPA hepatocyte uptake in healthy humans was reported as 0.08 ± 0.02 min^−1^ by Berks *et al*, 0.22 ± 0.05 min^−1^ by Georgiou *et al*, 0.29 ± 0.11 min^−1^ by Forsgren *et al*, 0.37 ± 0.19 min^−1^ by Dahlqvist Leinhard *et al*, and 3.4 ± 1.9 min^−1^ by Sourbron *et al*.^6,7,9,18,42^ Using the TRISTAN model values computed with LAR without preprocessing the data with a noise filter, we estimated uptake at 1.0 ± 0.3 min^−1^ in dogs and 0.24 min^−1^ for the pig. Human hepatocyte efflux rates for Gd-EOB-DTPA were reported as 0.02 ± 0.01 min^−1^ by Berks *et al*, 0.017 ± 0.006 min^−1^ by Georgiou *et al*, and 0.23 ± 0.17 min^−1^ by Forsgren *et al*.^6,18,42^ We estimated Gd-EOB-DTPA efflux at 0.027 ± 0.010 min^−1^ in dogs and 0.15 min^−1^ in the pig using the TRISTAN model with the LAR fitness criterion and no noise filtering. Although the TRISTAN model produced stable estimates for the influx and efflux of hepatospecific GBCAs, there was consistently a mismatch in the initial rise after bolus injection between the fitted model and empirical data, which suggests some dynamic phenomena in the images are not accounted for in this model.

Dahlqvist Leinhard *et al* reported the hepatocyte uptake rate for Gd-BOPTA in healthy humans as 0.03 ± 0.02 min^−1^.^7^ Using the TRISTAN model with LAR fitness criterion and no noise filtering, we estimated the Gd-BOPTA uptake rate in the pig at 0.21 min^−1^. Rapid efflux was observed in one Gd-BOPTA scan in which the pig was hyperthermic. This phenomenon is well known in mice, with higher temperature resulting in faster uptake and clearance of agents from the liver.^43^ Because of the hyperthermia, that scan was excluded from the study.

Impaired hepatocellular uptake is typical in liver disease.^2^ The large difference (≥ seven orders of magnitude) observed between TRISTAN k1 values for hepatospecific agents vs. non-hepatospecific agents in phenotypically normal dogs that are genetic carriers of GSD III and a normal pig is a promising indicator that this model shows the sensitivity to hepatocyte uptake required of a biomarker for liver function. It has also been suggested that the biliary efflux rate tracks fibrosis severity even in early liver disease in humans.^19^

The Berks model fit the shape of the liver concentration curves for both hepatospecific and non-hepatospecific agents well. In the pig, the $\alpha_{+}$ and $\alpha_{-}$ values show promise in distinguishing between hepatospecific and non-hepatospecific agents, but a larger sample size is needed to evaluate this statistically. The arterial flow fraction ($f_{a}$) values obtained were outside the physiologic range in most cases for hepatospecific agents in both the dogs and the pig. Application of the *post hoc* active uptake and efflux interpretation to the fitted parameters of the Berks model failed to produce physiologically meaningful parameters in many cases. For example, the derived extracellular space volume fraction values ($v_{\mathrm{ecs}}$) were invalid (> 1) in all cases. Thus, values of the active uptake and efflux interpretation are not presented here.

The analysis software we used was an independent implementation of pharmacokinetic models published by other groups. This replication contributes toward independent evaluation of these published models. Gabor and Banga advocated using global optimization methods for fitting pharmacokinetic models because these inverse problems are nonconvex and may be ill-conditioned, depending on the number of model parameters and quantity of experimental data available. Thus, local optimizers may converge on local solutions, leading to incorrect results.^44^ We employed a Multi-Start optimizer and carefully chosen ranges of the starting estimates with the goal of avoiding convergence to suboptimal parameter estimates for the pharmacokinetic models.

In most cases, there was no clear advantage in choosing one fitness criterion over another. This may be because the sample size is insufficient to reveal a difference. Nonetheless, because some numerically degenerate cases were encountered when using the LSQ fitness criterion to fit the TRISTAN model to scans performed with non-hepatospecific agents, it seems prudent to prefer the LAR criterion which did not exhibit this problem. No discernible advantage of fitting models to filtered vs. unfiltered data was identified. The small number of subjects may be insufficient to elucidate a pattern. Alternately, it is possible that a difference would be more noticeable if the scans were performed with a different temporal resolution or if smaller ROIs were used in the analyses.

When it occurs, overestimation of $f_{a}$ likely results from a low quality portal vein signal that causes the optimizer to find a better model fit by increasing the weighting of the abdominal aorta signal.^45^ Berks *et al* reported overestimation of $f_{a}$ in human volunteers and suggested that low temporal resolution, noise, and motion artifacts may be causative.^18^ Georgiou *et al* reported that $f_{a}$ was their least reproducible model parameter.^6^ Leporq *et al* reported that $f_{a}$ (referred to as “hepatic perfusion index” in their paper) increased with fibrosis severity.^19^ There’s no indication that any of our animals had fibrosis, but we lack histology data to definitively rule it out. We think it more plausible that the $f_{a}$ overestimates resulted from vascular input function data quality and limited temporal resolution rather than occult fibrosis in seemingly normal animals. Without some physiological rationale for the relationship between $f_{a}$ and fibrosis to differ between humans and other large animals, this suggests that $f_{a}$ determined using the Berks model may not be a reliable biomarker for fibrosis. The pharmacokinetic model used by Leporq *et al* included an additional parameter representing the backflux via MRP3 from the hepatocytes to the Space of Disse.^19^

These studies had several limitations to consider. *In situ* liver and blood relaxivity data was available for Gd-EOB-DTPA^31^ and Gd-DTPA^40^ but unavailable for Gd-BOPTA and Gd-BT-DO3A. Measuring their *in situ* relaxivities may improve the accuracy of estimated pharmacokinetic parameters. These studies were intentionally designed as feasibility investigations and therefore involved a small sample size: three dogs scanned three times with a hepatospecific agent and once with a non-hepatospecific agent, and one pig scanned twice each with two hepatospecific agents and once with a non-hepatospecific agent. While this precludes population-level inferences, it was sufficient to assess model behavior, reproducibility, and translational plausibility. Although normal dogs would have been a preferable cohort for the canine study, the animals available to us were obtained from another scientist’s colony comprised of dogs that had surgically-corrected cleft palate and are genetic carriers of GSD III but exhibited no signs of the disease. This reflects the financial and logistical challenges associated with large animal studies as well as alignment with ethical standards regarding reuse of research animals. We aimed to achieve balance between temporal resolution, sufficient spatial resolution to image intravascular blood without confounding partial volume effects, and multi-slice coverage of relevant anatomy. However, the temporal resolution achieved (7.65 s for dogs and 13.4 to 13.5 s for the pig) did not enable visualization of first pass dynamics. Additionally, we fixed the time delay value for vascular inputs to the liver to zero for the Berks model, as the expected time delay was too small relative to the temporal resolution to permit accurate determination as a free parameter. Lastly, the study scope was limited to scanning healthy animals only. Thus, the pharmacokinetic models’ utility as a biomarker for disease status was not evaluated. This remains an area of interest for future studies.

## Conclusions

DCE-MRI of dogs and a pig revealed rapid distribution in the blood and high accumulation in the liver shortly after IV injection of hepatospecific MRI contrast agents Gd-EOB-DTPA and Gd-BOPTA. There was no accumulation of the non-specific agents Gd-DTPA or Gd-BT-DO3A in the liver. Proper thermoregulation of the animal during imaging seems essential for accurate and reproducible results. We successfully fit two different, previously validated, pharmacokinetic models to the imaging data, with results showing that the kinetic parameters are similar enough to humans to encourage further study of either of these two large animal models for translational research. Despite the modest number of animals, the reproducibility and magnitude of the observed effects indicate that meaningful quantitative insights can be obtained from well-controlled feasibility studies. These findings demonstrate that carefully designed small-cohort experiments can establish baseline model parameters and guide subsequent translational work. Given the lack of hepatic uptake of Gd-BOPTA in humans, we suggest Gd-EOB-DTPA should be used in dog and pig models for biomedical imaging.

## Supplementary Material

Supplementary Information:

Supplementary Figure 1 is an additional figure constructed in the same manner as Figure 3 but showing the data without noise filtering.

Supplementary Files

This is a list of supplementary files associated with this preprint. Click to download.

• Table1.pdf

• Table2.pdf

• Table3.pdf

• Table4.pdf

• Table5.pdf

• 154096834616193coidisclosureICMJEMunoz.pdf

• 154096834616193coidisclosureICMJEforES.pdf

• 154096834616193coidisclosureICMJEforMTL.pdf

• COIHixJeremy.pdf

• ElectronicSupplementaryMaterial.docx

• Jiecoi.pdf

• Mallettdisclosure.pdf

• SupplementalFigure.pdf

## Figures and Tables

**Table 1.** Contrast Agent Information And Dosing For Swine MRI Studies The order of the MRI scans and associated contrast agent, animal weight, and dosing information is shown for the swine study.

**Table 2.** Contrast Agent Information And Dosing For Canine MRI Studies The order of the MRI scans and associated contrast agent, animal weight, and dosing information is shown for the canine study.

**Table 3.** MR Acquisition Parameters The pulse sequence and corresponding acquisition parameters utilized for the dynamic contrast-enhanced MRI scans for dogs and the pig are shown.^50,51^ All scans were performed at MSU Veterinary Medicine using their Siemens Espree 1.5 T scanner.

**Table 4.** Estimated Model Parameters — Swine Study A reference region model (TRISTAN model) and a biexponential model using dual vascular input functions (Berks model) were fit to whole liver ROI signals for each pig MRI scan. Scans were performed twice with Gd-EOB-DTPA, twice with Gd-BOPTA, and once with Gd-DTPA. The pig was hyperthermic during the first Gd-BOPTA scan and, therefore, that data was excluded from the analysis. The spleen reference region was not present within the FOV for one of the Gd-EOB-DTPA scans. Thus, the TRISTAN model could not be fit for that particular scan. Model fitting was performed four times per model per MRI scan, using LAR and LSQ fitness criteria with and without pre-processing the data with a noise filter. Where more than one scan was analyzed, the model parameter estimates are reported as the mean ± SD.

**Table 5.** Estimated Model Parameters — Canine Study A model (TRISTAN) using the spleen as a reference region and a biexponential model (Berks) using the abdominal aorta and portal vein signals, respectively, as its input functions were fit to the liver ROI signal for each MRI scan of the dogs. Scans were performed three times with Gd-EOB-DTPA and once with Gd-BT-DO3A for each dog. Model fitting was performed four times per model per MRI scan, using LAR and LSQ fitness criteria with and without pre-processing the data with a noise filter. The model parameter estimates are reported as the mean ± SD.

## Data Availability

Imaging data is available via a public repository.^46^

## Software Availability:

Software entitled LiverDCE was implemented using MATLAB^®^ (RRID:SCR_001622, R2023a) to perform the pharmacokinetic analyses. Source code is available via a public repository.^41^ Program dependencies include the Medical Imaging, Optimization, Global Optimization, Image Processing, Parallel Computing, Curve Fitting, Signal Processing, Statistics and Machine Learning, and Symbolic Math toolboxes for MATLAB^®^; afni_matlab^47^; dicm2nii^48^; and a modified version of imtool3D^49^.
